# BIS-Guided Total Intravenous Anesthesia for Orchiopexy and Circumcision in a Child with Severe Autism: A Case Report

**DOI:** 10.1155/2012/718594

**Published:** 2012-11-21

**Authors:** Selçuk Okur, Müge Arıkan, Gülşen Temel, Volkan Temel

**Affiliations:** Department of Anesthesiology, Karabük State Hospital, Karabük, Turkey

## Abstract

Autistic children are very difficult to manage in the hospital setting because they react badly to any change in routine. We describe a case of 10-year-old male patient with severe autism undergoing orchidopexy and circumcision. Following premedication, anesthesia was induced with remifentanil, propofol, atracurium, and maintained with total intravenous anesthesia (propofol and remifentanil). The Bispectral Index System was monitored for determination of the depth of anesthesia. After surgery, all infusions were discontinued. The patient was then transferred to the postanesthetic care unit. There were no adverse events observed during the anesthetic management. The patient was discharged from the hospital on the second postoperative day. Bispectral Index System-guided Total Intravenous Anesthesia can provide some advantages for patient with autism, such as hemodynamic stability, early and easy recovery, to facilitate faster discharge, to optimize the delivery of anesthetic agents, to minimize its adverse effects, and to maximize its safety.

## 1. Introduction

Autism is an heterogeneous developmental disorder mainly characterized by three domains of impairments: communication-language, social interaction, and behavioral oddities [1]. There is a great variation in the severity of autism and hospital needs of these children. Patients with impaired ability to understand and communicate can be difficult to manage perioperatively [2, 3]. Various agents and methods have been tried in these patients. Autistic patients scheduled to surgical procedures demand special care, such as detailed preanesthetic evaluation with history and specific physical evaluation and careful choice of anesthetic technique [2]. There appears to be little literature in paediatric anaesthetic practice relevant to children suffering with autism. Recent findings suggest a need for rigorous study of the potential problems that autistic children may have when undergoing an anaesthetic [4]. 

Patients with autism present many potential problems in terms of management of anesthesia. This case aimed at reporting a case of Bispectral Index System-guided TIVA in autistic patient.

## 2. Case Report

After his family written consent, a 10-year-old male child (32 kg), classified as American Society of Anesthesiologists physical status III (with severe autism, mild mental retardasyon, language disability-stage 5, musculo-skeletal and nervous system weakness, and hearing loss in the left ear). Preoperative laboratory tests were abnormal. Patient was scheduled for orchidopexy and circumcision under general anesthesia.

Upon arrival in the operating room, standard monitoring, including EKG, pulse oximetry, end-tidal capnography, Bispectral Index (BIS vista, Aspect Medical Systems, The Netherlands), and Train-of-four monitor (TOF watch, Organon, Ireland) were applied. Preoperative vital signs included blood pressure of 86/52 mmHg, a heart rate of 130 beats/min, oxygen saturation rate of 98%, and sinus rhythm on the electrocardiogram.

 After establishing an intravenous access, he was given midazolam 1 mg (iv). The patient's fluid level was maintained with crystalloid solution. Anaesthesia was induced with remifentanil (1 *μ*g/kg), propofol (2 mg/kg). Atracurium (0.5 mg/kg) was used to intubate the patient. After to reach Train-of-four ratio (T4/T1) at 0, he was intubated with an internal diameter 5.5 mm endotracheal tube under direct laryngoscopy. No problems were encountered during tracheal intubation and the breathing sound was clear at both lung fields. The patient was mechanically ventilated in the pressure control mode, and the end-tidal carbon dioxide levels were monitored and maintained at the range of 30–32 mmHg. Anesthesia was maintained with oxygen (1 L/min), medical air (1.5 L/min), and continuous infusion of 100–150 *μ*g/kg/min of propofol and 0.2–0.4 *μ*g/kg/min of remifentanil using an infusion pump (AS50 Auto Syringe Infusion Pump, Baxter International Inc. Deerfield, IL, USA). The infusions of propofol and and remifentanil were adjusted in order to keep BIS as  50 ± 10. To maintain T1 in the TOF ratio (T4/T1) at 25%, atracurium (0.2 mg/kg) was intravenously injected with the top-up dose. 

Oxygen saturation, heart rate, noninvasive hemodynamometer, and BIS values were recorded at 5 min intervals intraoperatively. Vital signs were stable throughout the procedure. Total procedure time was 45 min. After skin closure, all infusion drugs were stopped and antagonism of residual neuromuscular block was carried out by 35 *μ*g/kg neostigmine together with 20 *μ*g/kg atropine when T4/T1 ratio reached 75% or higher followed by tracheal extubation (3.51 min). The time from stoppage of propofol and remifentanil infusions until BIS level raised to 80 was considered as the recovery time (6 min). The patient was then transferred to the postanesthetic care unit. The patient was discharged from the hospital on the second postoperative day.

## 3. Discussion

Mental disability is a term which is used when an individual's intellectual development is significantly lower than average, limiting his or her ability to adapt to the environment [5]. Autism is a neurobehavioral and cognitive disorder characterized by impaired development of interpersonal and communication skills, limited interests, and repetitive behaviors. The incidence of autism is about 0.2% [6]. There are enormous variations in the behavioral patterns and the severity of illness among individuals with autism. Mental retardation is evident in approximately 70% of individuals with autism. The behavioral symptoms in children include temper tantrums, hyperactivity, short attention span, impulsivity, agitation, anger, and a tendency for aggressive and self-injurious behaviors [7]. Disorders of language and social communication, poor response to external stimulation, tendency to isolate themselves, and poor eye-to-eye contact are well-recognized symptoms. 

Patients with impaired ability to understand and communicate can be difficult to manage perioperatively. They frequently require lateral thinking on the part of the anesthesiologists to make the induction process as smooth as possible. The main targets of these patients are rapid recovery, smooth postoperative pain, early discharge and low stress during the peroperative period [8].

Solak et al. reported that TIVA can be valuable alternative to inhalation anesthesia for children [9]. The bispectral index is a pharmacodynamic measure of the effect of anesthesia on the central nervous system. Messieha et al. announced that monitoring anesthesia with BIS promotes earlier extubation and discharge for pediatric dental patients who receive general anesthesia [10]. Park et al. aimed that their study was to investigate the relationship between BIS index and predicted plasma concentration of propofol delivered by target-controlled infusion during emergency in children. As a result they concluded that when respiration returned, mean BIS was 77.2 ± 5.3 and propofol plasma concentration 1.6 ± 0.3 *μ*g/kg and when a verbal command was obeyed, BIS was 82.4 ± 5.6 and propofol plasma concentration 1.5 ± 0.3 *μ*g/kg and BIS moderately correlated with the predicted plasma concentration of propofol [11]. Zhang et al. reported that BIS effectively monitors the depth of intravenous anesthesia with remifentanil and propofol [12].

Asahi et al. suggested that autistic patients have greater propofol requirements for anaesthesia during ordinary dental treatment compared with intellectually impaired patients [13].

In our patient, anesthesia was induced with propofol and remifentanil and maintained with propofol and remifentanil infusions. The dosages of propofol and remifentanil were adjusted in order to keep BIS as 50 ± 10. Vital signs were stable throughout his procedure.

Bispectral Index System-guided TIVA can provide some advantages for patient with autism, such as hemodynamic stability, early and easy recovery, to facilitate faster discharge, to optimize the delivery of TIVA, to minimize its adverse effects, and to maximize its safety.
